# Prominent differences in left ventricular performance and myocardial properties between right ventricular and left ventricular-based pacing modes in rats

**DOI:** 10.1038/s41598-017-06197-w

**Published:** 2017-07-19

**Authors:** Wesam Mulla, Sharon Etzion, Sigal Elyagon, Roni Gillis, Michael Murninkas, Yuval Konstantino, Ingra Mannhardt, Thomas Eschenhagen, Noah Liel-Cohen, Yoram Etzion

**Affiliations:** 1 Cardiac Arrhythmia Research Laboratory, Department of Physiology and Cell Biology, Faculty of Health Sciences, Ben-Gurion University of the Negev, Beer-Sheva, Israel; 2 Regenerative Medicine & Stem Cell Research Center, Ben-Gurion University of the Negev, Beer-Sheva, Israel; 3 Cardiology Department, Soroka University Medical Center and the Faculty of Health Sciences, Ben-Gurion University of the Negev, Beer-Sheva, Israel; 4 Department of Experimental Pharmacology and Toxicology, Cardiovascular Research Center, University Medical Center Hamburg-Eppendorf, Hamburg, Germany; 5 DZHK (German Center for Cardiovascular Research), partner site Hamburg/Kiel/Lübeck, Hamburg, Germany

## Abstract

Biventricular pacing is an important modality to improve left ventricular (LV) synchronization and long-term function. However, the biological effects of this treatment are far from being elucidated and existing animal models are limited and demanding. Recently, we introduced an implanted device for double-site epicardial pacing in rats and echocardiographically demonstrated favorable effects of LV and biventricular (LV-based) pacing modes typically observed in humans. Here, this new animal model was further characterized. Electrodes were implanted either on the right atria (RA) and right ventricle (RV) or on the RV and LV. Following recovery, rats were either used for invasive hemodynamic measurements (pressure-volume analysis) or exposed to sustained RV vs. biventricular tachypacing for 3 days. RV pacing compromised, while LV-based pacing modes markedly enhanced cardiac performance. Changes in LV performance were associated with prominent compensatory changes in arterial resistance. Sustained RV tachypacing increased the electrocardiogram QTc interval by 7.9 ± 3.1 ms (n = 6, p < 0.05), dispersed refractoriness between the right and left pacing sites and induced important molecular changes mainly in the early-activated septal tissue. These effects were not observed during biventricular tachypacing (n = 6). Our results demonstrate that the rat is an attractive new model to study the biological consequences of LV dyssynchrony and resynchronization.

## Introduction

The electromechanical and molecular consequences of various ventricular pacing modes have become a subject of intense interest in recent years due to profound clinical implications^1–3^. Compelling evidence indicates that right ventricular (RV) pacing can be detrimental to left ventricular (LV) function. The sequence of electrical activation during RV pacing may largely resemble the activation pattern of left bundle branch block (LBBB)^4^, and can result in dyssynchronous LV contraction^5, 6^. In accordance, RV pacing increases the risk for heart failure (HF) even in patients without pre-existing LV dysfunction^7–10^.

Cardiac resynchronization therapy (CRT) by means of biventricular (BiV) pacing was developed as a strategy to overcome the deleterious effects of electromechanical dyssynchrony in the failing heart and represents a major advance in HF therapy^11^. BiV pacing has been demonstrated to improve contractile performance in patients with dyssynchrony and to improve long-term survival in selected patients^2^. Although the clinical effectiveness of CRT is remarkable, there are multiple questions concerning dyssynchrony and CRT that remain ill-defined at present. For example, patient vulnerability to HF progression as a result of iatrogenic RV pacing is variable and optimal management of this problem is controversial^12, 13^. In addition, it is estimated that around 30% of selected patients based on current indications do not benefit from CRT. Clinical data indicate that improvement in dyssynchrony is a prerequisite for obtaining beneficial effects of CRT, but the extent of such improvement may correlates poorly with clinical outcomes^14^. Although detailed evaluation including assessment of RV dyssynchrony may supplement LV dyssynchrony information and improve outcome prediction^15^, responsiveness to CRT also appears to involve factors beyond mechano-energetic considerations^2, 3^. Recent studies exploring the cellular and molecular impacts of CRT in the failing heart indicate a highly complicated picture of cellular and molecular sequence of events, which is still far from being elucidated and which might be the key for better treatment adjustment and prediction of outcome^3, 16–19^. Thus, reliable models that support extensive research of the complex biology of dyssynchrony and resynchronization are of paramount importance for obtaining new clinical insights in this field.

Current animal studies dealing with dyssynchrony and resynchronization almost exclusively utilize large animals (mainly dogs). Although insights coming from such models are extremely valuable, their highly demanding nature restricts usage for proof-of-principle studies and widespread experimentation at multiple laboratories^20, 21^. In addition, the ability to induce clinically relevant manipulations such as hypertension, diabetes as well as molecular and genetic modifications, are quite limited in large animal models. While rodents have many such advantages and are already extensively used in HF research^22^, technical difficulties in electrode implantation have largely prevented proper validation of the effects of various pacing modes in rodents. Two murine studies utilizing single site pacing of RV and M-mode echocardiography have managed to clearly demonstrate dyssynchrony between the septum and the LV lateral wall^23, 24^. However, single site ventricular pacing is methodologically problematic since it requires comparison to the spontaneous rhythm, which is different from the pacing state not only in ventricular electromechanics but also in beating rate and atrio-ventricular synchrony. Recently, using double site bipolar electrode implantation in combination with electrical recordings and speckle-tracking echocardiography, we managed to characterize for the first time the effects of various ventricular pacing modes on LV electromechanical synchrony in rats^25^. We found that RV pacing induces marked LV dyssynchrony compared to right atrial (RA) pacing or sinus rhythm. In contrast, LV pacing and, to a greater extent BiV pacing, diminished LV dyssynchrony^25^. These findings support the notion that rodent cardiac pacing mimics important electromechanical features seen in humans. However, the actual effects of different pacing modes on LV performance and on the myocardial properties of the rodent heart are still unknown. Therefore, in the present study we used pressure-volume (PV) loop recordings to characterize the effects of different ventricular pacing modes on cardiac performance. Next, we compared myocardial properties following application of sustained RV or BiV tachypacing for 72 hours in conscious freely moving rats. Our findings indicate similarities in the effects of various pacing modes to humans, but also demonstrate remarkable effects on arterial elastance, which were not described in humans to a similar extent. In addition, we found that sustained RV tachypacing induces prominent electrical and biochemical changes in myocardial properties compared to BiV pacing, including the activation of JNK in the early-activated myocardium, which was not previously identified in this setting. Overall, our data mark this model as an attractive new tool to study the complex pathophysiology of ventricular dyssynchrony and resynchronization.

## Results

### Hemodynamic effects of RV pacing-induced dyssynchrony

**Figure 1:**
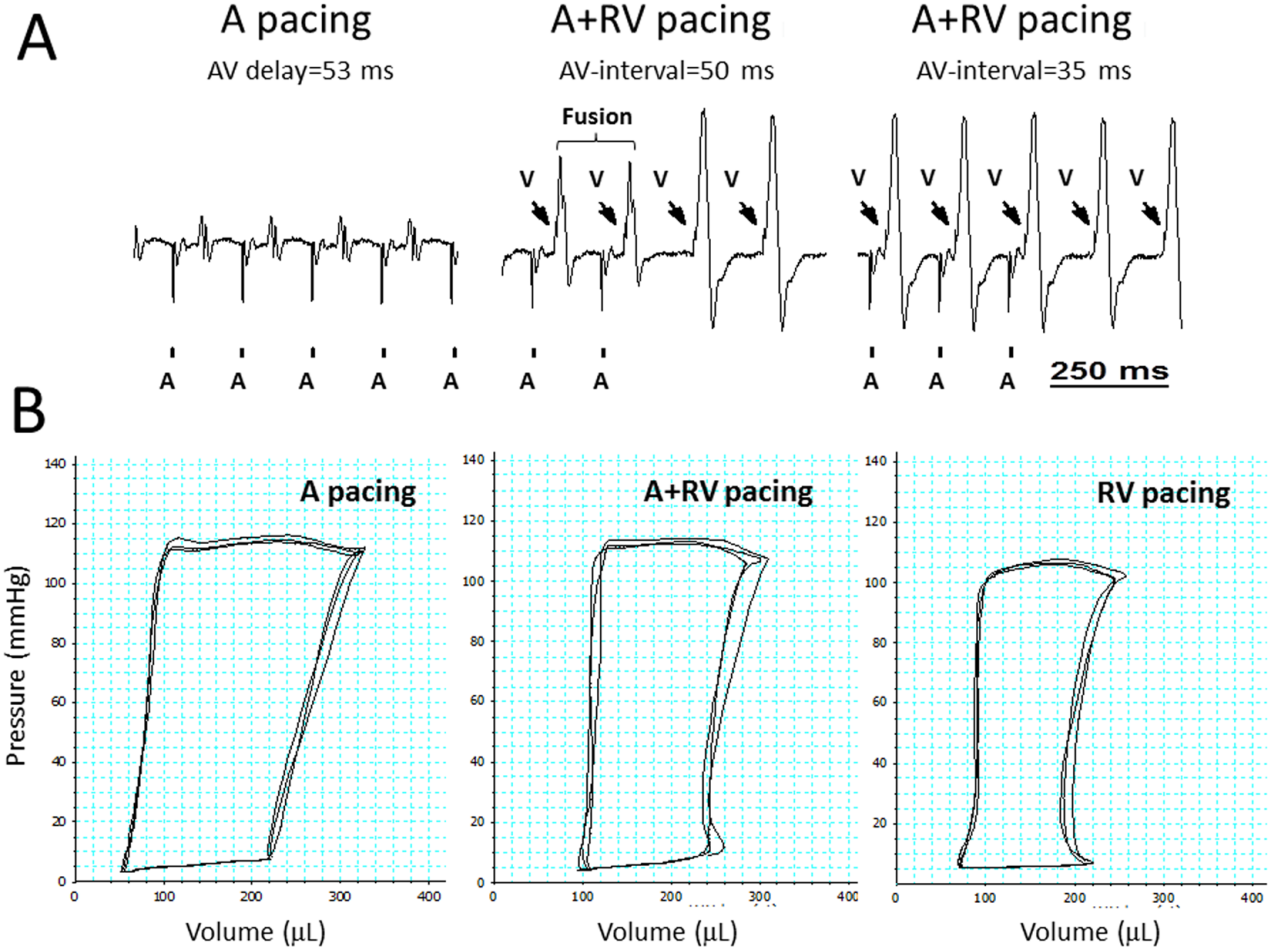
RV pacing-induced decrease in LV hemodynamic performance is related to both dyssynchrony and impaired AV coupling. Analysis of rats implanted with an atrial (**A**) bipolar electrode and a right ventricular (RV) bipolar electrode for sequential A + RV pacing. (**A**) ECG recording (lead I) during: *Left*; override A pacing. *Mid*; override A + RV pacing (AV-interval = 50 ms) or RV-only pacing. *Right*; override A + RV pacing (AV-interval = 35 ms) or RV-only pacing. Vertical bars at the bottom indicate A pacing; arrows indicate RV pacing. Note fusion beats with an AV interval of 50 ms and absence of fusion beats when the AV interval was reduced to 35 ms. (**B**) PV-loop recordings during A pacing, A + RV pacing (using the maximal AV-interval that did not induce fusion beats) and RV-only pacing. Comparison of A vs. A + RV pacing shows the effects of LV dyssynchrony, while RV-only pacing also induces preload reduction due to impaired AV coupling.

In the absence of technical means to induce LBBB, iatrogenic induction of electromechanical dyssynchrony relies on overdrive RV pacing in our rat model^25^. Thus, it is imperative to characterize in details the hemodynamic effects of RV pacing as compared to normally conducted, synchronous contraction. To enable such analysis we implanted pacing electrodes on both the RA and the RV and utilized them to induce either overdrive atrial (A) pacing (synchronous) or sequential atrial and RV (A + RV) pacing (dyssynchronous) with similar beating rate and atrioventricular (AV) coupling. Using conventional ECG analysis we adjusted the AV interval during A + RV pacing to achieve complete RV pacing activation sequence (Fig. 1A). The mean AV interval fulfilling such conditions was 39.4 ± 3.2 ms (n = 8).

P-V loop recordings during A pacing and A + RV pacing did not show an effect of dyssynchrony on LV loading, as inferred from the similar EDV and EDP of the two pacing modes (Table 1). However, A + RV pacing markedly reduced LV systolic parameters including Pdev, SV, EF, SW and + dP/dt max (Fig. 1B and Table 1). These effects largely resemble the hemodynamic effects of LBBB or iatrogenic RV pacing in large mammalian hearts^26–29^. In addition, A + RV pacing also reduced dP/dt min and prolonged Tau, indicating that pacing- induced dyssynchrony slows relaxation and impairs the diastolic function of the LV, again in a remarkable resemblance with findings in large mammalian hearts^30^. Application of RV-only pacing had additional effects consistent with reduced LV loading, including reduced SV and Pdev that we attribute to lack of physiological AV synchrony during ventricular overdrive pacing (Fig. 1B and Table 1).

**Table 1:** PV loop analysis of rats implanted with RA and RV pacing electrodes.

Parameter	A pacing	A + RV pacing	RV pacing	P value
**Pacing CL** (ms)	150	150	150	—
**Pmean** (mm Hg)	59.84 ± 2	59.86 ± 1.8	51.4 ± 2.3^#,^^	<0.001
**Pdev** (mm Hg)	118.9 ± 4.1	114.2 ± 4.3*	105.6 ± 4^#,^^	<0.001
**ESP** (mmHg)	104.3 ± 8	109.4 ± 4.5	100.8 ± 4.5^#,^^	0.008
**EDP** (mmHg)	5.4 ± 0.5	5.9 ± 0.5	5.7 ± 0.8	NS
**ESV** (μL)	51.1 ± 11.4	81.8 ± 9.2*	59.1 ± 10.1^#^	<0.001
**EDV** (μL)	283.4 ± 18.1	286.9 ± 16.4	233.5 ± 22.1^#,^^	<0.001
**SV** (mL)	232.4 ± 8.5	205.1 ± 9.6*	174.4 ± 13.8^#,^^	<0.001
**Ea** (mm Hg/μL)	0.45 ± 0.03	0.54 ± 0.02*	0.59 ± 0.04^^^	0.004
Systolic indices	—	—	—	—
**EF** (%)	83.3 ± 3.2	72.0 ± 2.3*	75.9 ± 2.9^^^	<0.001
**SW** (mm Hg*μL)	21104 ± 1074	17061 ± 1810*	13356 ± 1767^#,^^	<0.001
**dP/dt max** (mm Hgs^−1^)	8737.9 ± 406	7048 ± 415*	6639.0 ± 433^^^	<0.001
Diastolic indices	—	—	—	—
**dP/dt min** (mm Hgs^−1^)	−8076 ± 310.4	−6407.6 ± 402.1*	−6837.9 ± 359.5^^^	<0.001
**Tau** (ms)	9.87 ± 0.32	12.14 ± 0.46*	10.77 ± 0.38^#,^^	<0.001

Repeated P-V loop measurements in rats under atrial (A), sequential atrial and RV (A + RV) and RV only pacing modes (n = 8 for all). Statistical analysis was performed using non-parametric repeated measure ANOVA (Friedman test). Symbols indicate significant difference in post-test analysis; *RA + RV vs. RA (ventricular dyssynchrony component), ^#^RV vs. RA + RV (AV physiology component), ^^^RV vs. RA (combined ventricular dyssynchrony and AV physiology components). **Pmean** = mean pressure; **Pdev** = developed pressure; **ESP**-end systolic pressure; **EDP**-end diastolic pressure; **ESV** = end systolic volume; **EDV** = end diastolic volume; **SV** = stroke volume; **Ea** = arterial elastance; **EF** = ejection fraction **SW** = stroke work.

An additional important finding was a prominent increase of Ea during A + RV pacing and a further increase during RV-only pacing (Table 1). Since pacing rate was the same in all measurements, this result directly reflects augmentation of net arterial load that is imposed on the LV^31^. Of note, increased Ea is not typically observed in humans exposed to dyssynchronous pacing^32^ and seems to reflect compensatory changes in the highly dynamic vascular tone of rodents (see discussion). Moreover, in humans a reduction of Ea during RV pacing might be associated with pacing-induced mitral regurgitation^33^. Thus, the elevated Ea we observed during RV pacing implies absence of RV pacing-induced mitral regurgitation in our model. Indeed, we did not detect mitral retrograde flow waves during any of the overdrive pacing modes (n = 5). In addition, we did not notice acute changes in left atrial diameter between the different ventricular pacing modes (n = 4, not shown).

### Hemodynamic effects of RV pacing vs. LV-based pacing

**Figure 2:**
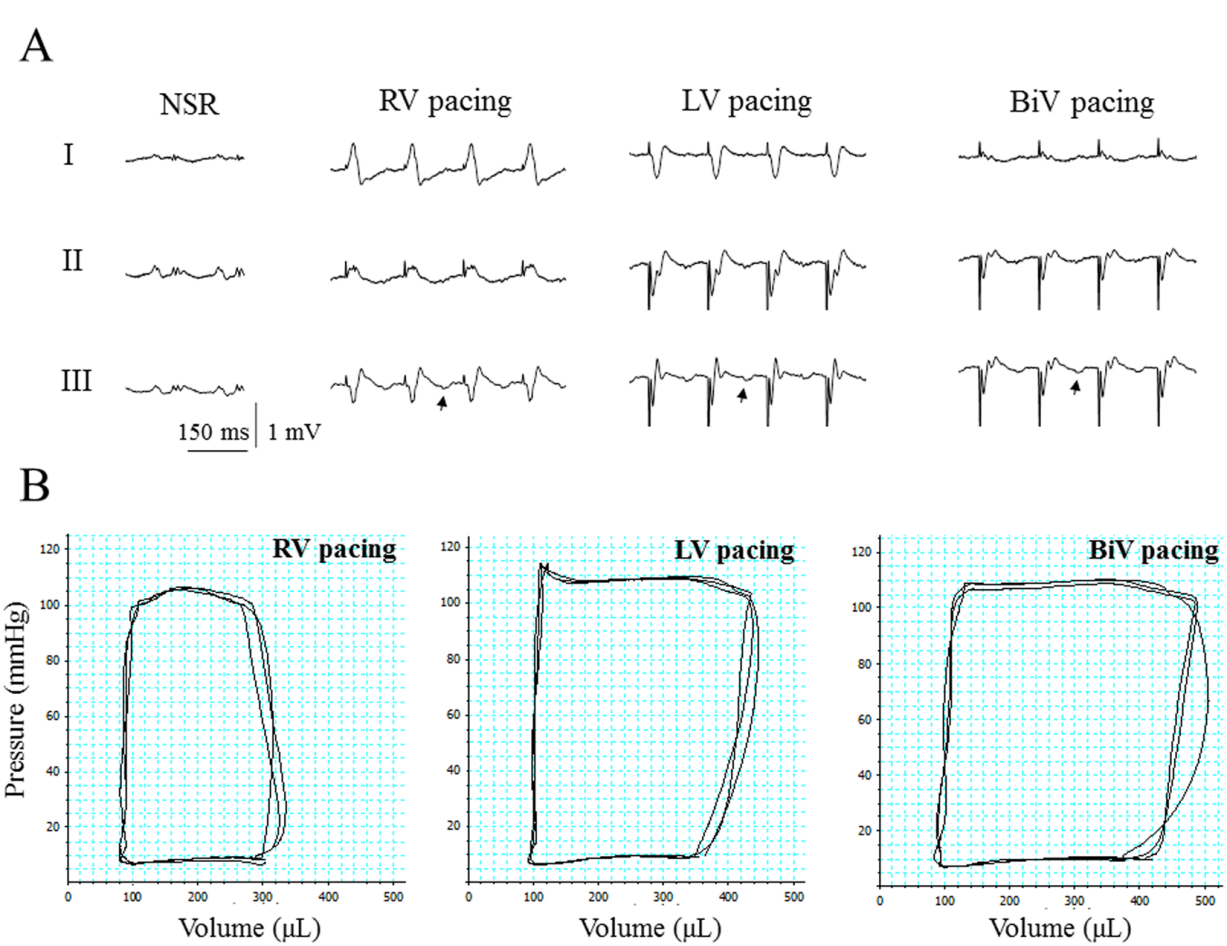
LV-based pacing modes markedly improve LV hemodynamic performance compared with RV pacing. Analysis of rats implanted with RV bipolar electrode and LV bipolar electrode. (**A**) ECG recordings during normal sinus rhythm (NSR) and the various ventricular pacing modes. Note LBBB like QRS pattern during RV pacing and a remarkable QRS shortening during BiV pacing. Note similar timing and morphology of the retrograde p-waves (marked by arrows), indicating that the component of AV-dyssynchrony is similar in all three ventricular modes of pacing. (**B**) PV-loop recordings during RV pacing, LV pacing and BiV pacing. Note the profound effects of LV pacing and BiiV pacing in comparison with RV pacing.

Following the above analysis of RV pacing-induced dyssynchrony relative to synchronous pacing, we next used implanted RV and LV pacing electrodes to compare the hemodynamic effects of various ventricular pacing modes. ECG recordings confirmed the previously described effects on QRS duration and morphology^25^. A typical example of obtained electrocardiograms is shown in Fig. 2A with LBBB like pattern during RV pacing and an opposite QRS vector with different morphology during LV pacing. BiV pacing produced a QRS morphology that was distinct from that observed with single chamber stimulation of either ventricle. The recordings also confirm the existence of similar retrograde P waves in all modes of ventricular pacing, an important methodological consideration for proper comparison between various ventricular pacing modes.

P-V loop recordings indicated profound differences in LV hemodynamic performance, when LV and BiV pacing were compared with RV pacing (Fig. 2B and Table 2). LV systolic function indices were significantly improved acutely during LV-based pacing modes compared with RV pacing. In contrast, indices related to LV diastolic function did not differ significantly between the three pacing modes. The finding that LV EDV was higher while LV EDP remained the same during LV-based pacing modes strongly suggests that the external constraint to LV filling was ameliorated compared with RV pacing (see discussion). In accordance with the augmentation of Ea induced by RV pacing (Table 1), LV-based pacing modes markedly decreased Ea (Table 2) by ~43%, suggesting compensatory changes of arterial resistance in response to the enhanced cardiac performance during LV-based pacing.

**Table 2:** PV loop analysis of rats implanted with RV and LV pacing electrodes.

Parameter	RV pacing	LV pacing	BiV pacing	P value
Pacing CL (ms)	150	150	150	—
**Pmean** (mm Hg)	45.1 ± 1.8	46.3 ± 1.9	49.3 ± 1.3^#,^^	0.005
**Pdev** (mm Hg)	93.3 ± 2.7	95.5 ± 2.4	99.6 ± 2.0^#,^^	0.002
**ESP** (mmHg)	86.7 ± 4.6	81.7 ± 6.3	84.6 ± 6.1	NS
**EDP** (mmHg)	8.3 ± 1	7.4 ± 1.1	8.5 ± 1.1	NS
**ESV** (μL)	76.1 ± 24.3	85.1 ± 23.8	82.7 ± 22.1	NS
**EDV** (μL)	288.9 ± 34.6	401.5 ± 17.8*	438.5 ± 29.1^#^	<0.001
**SV** (mL)	212.6 ± 22.4	316.4 ± 22.5*	355.7 ± 35.7^#^	<0.001
**Ea** (mm Hg/μL)	0.46 ± 0.07	0.27 ± 0.03*	0.26 ± 0.03^#^	<0.001
Systolic indices	—	—	—	—
**EF** (%)	75.3 ± 4.8	79.2 ± 4.8	80.7 ± 4.8	NS (0.07)
**SW** (mm Hg*μL)	16084 ± 2008	24912 ± 2187*	27393 ± 2721^#^	<0.001
**dP/dt max** (mm Hgs^−1^)	5747.1 ± 389.7	6433.6 ± 271.6*	6824.3 ± 345.9^#,^^	<0.001
Diastolic indices	—	—	—	—
**dP/dt min** (mm Hgs^−1^)	−5394.6 ± 305.2	−5476.1 ± 275.2	−5777.6 ± 223.6	NS
**Tau** (ms)	10.2 ± 0.46	10.41 ± 0.47	10.38 ± 0.55	NS

Repeated P-V loop measurements in rats under RV, LV and BiV pacing modes (n = 8 for all). Statistical analysis was done using repeated measure one way ANOVA. Symbols indicate significant difference in post-test analysis; *LV vs. RV, ^#^BiV vs. RV, ^^^BiV vs. LV. **Pmean** = mean pressure; **Pdev** = developed pressure; **ESP**-end systolic pressure; **EDP**-end diastolic pressure; **ESV** = end systolic volume; **EDV** = end diastolic volume; **SV** = stroke volume; **Ea** = arterial elastance; **EF** = ejection fraction **SW** = stroke work.

In an attempt to further clarify the mechanisms leading to the marked differences in LV performance between RV pacing and LV-based pacing modes, inferior vena-cava occlusion experiments were performed. Under our standard experimental conditions without mechanical ventilation, these attempts were challenging and largely failed to induce meaningful curves that could be properly analyzed. Supplemental Fig. 1S-A, demonstrates a successful attempt, which supports the notion that load-independent systolic function is enhanced during BiV pacing compared to RV pacing. Further attempts to get reliable data by applying mechanical ventilation and muscle relaxation were technically successful (Supplemental Fig. 1S-B), but yielded inconsistent results with regard to load-independent systolic and diastolic functions. Overall, although a tendency of increase was noted in preload recruitable SW and Cardiac efficiency during BiV pacing (Supplemental Table 2S) statistical analysis did not demonstrate significant differences in any of the examined parameters. Thus, we could not obtain definite conclusions regarding the mechanisms leading to increased cardiac performance during LV-based pacing at present.

### Electrophysiological consequences of sustained RV pacing vs. BiV pacing

Following detailed analysis of the acute consequences of various pacing modes, we next utilized our model to examine for the first time, the effects of sustained RV tachypacing vs. BiV tachypacing in freely moving rats. At baseline, there were no differences between the RV-paced and BiV-paced groups in any of the measured electrophysiological parameters including spontaneous corrected QT interval (QTc-NSR), paced QT interval during RV site pacing and LV site pacing, epicardial activation time during pacing of both sites and VERP during pacing of both sites.

**Figure 3:**
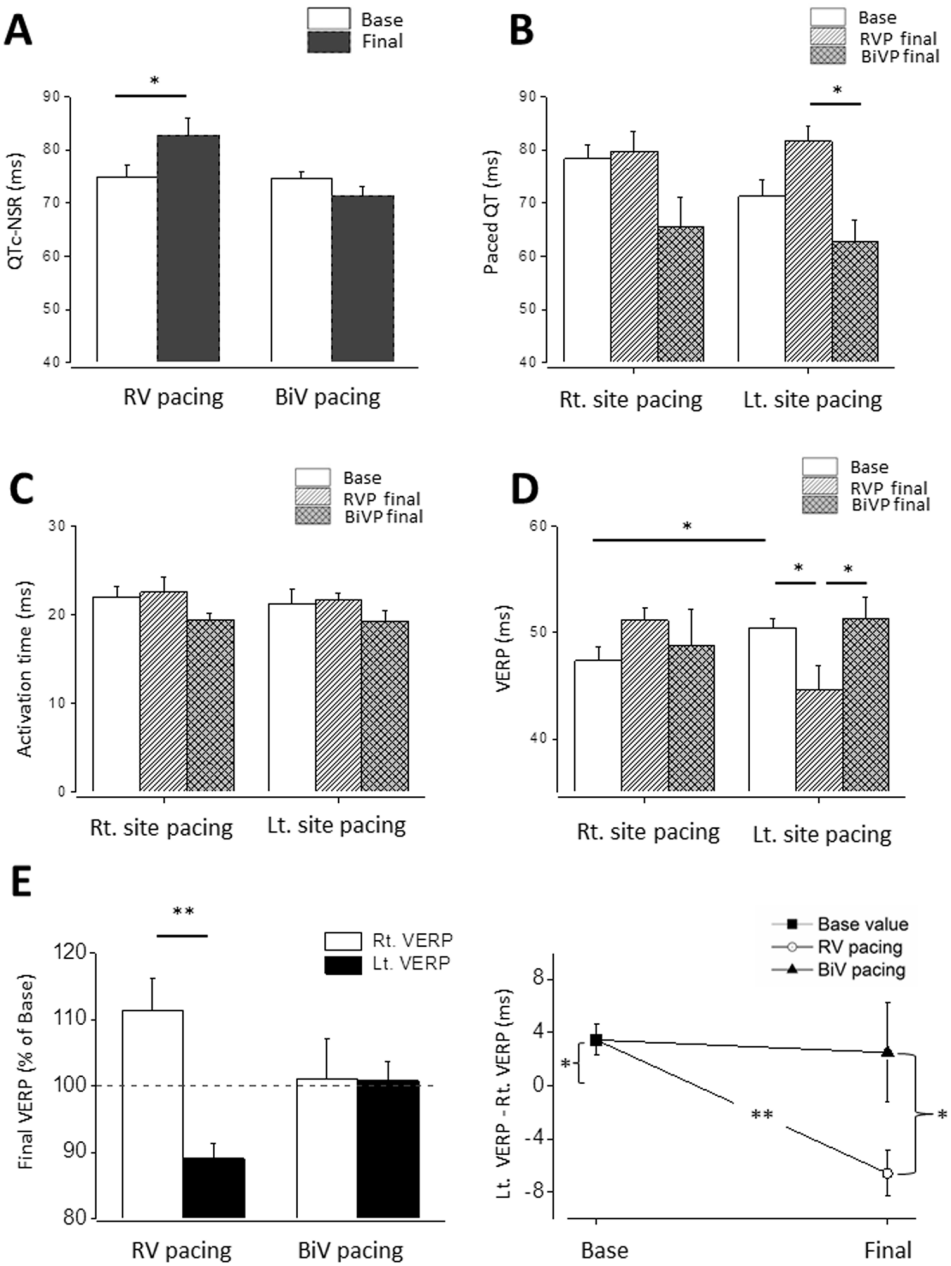
RV tachypacing prolongs native QTc interval and induces dispersion of VERP between the pacing sites in contrast to BiV tachypacing. Rats were implanted with RV pacing electrode and LV pacing electrode. Following a recovery period (7 d), rats were subjected to 72 h of either RV pacing or BiV pacing at a CL of 100 ms. (**A**) Measured QTc interval under sinus rhythm was evaluated in the conscious state using subcutaneous electrodes implanted on the back of the animals. Note prolongation of the QTc interval during RV tachypacing which did not occur in response to BiV pacing. Of note, statistical findings of QT and QTc measurements were similar (not shown), since the spontaneous heart rate of the rats did not change significantly by the pacing. (**B**) Paced QT interval following RV vs. BiV tachypacing. Following 72 h of pacing there was a significant difference between two ventricular pacing groups during Lt. site pacing. (**C**) Epicardial activation during Rt. site pacing and Lt. site pacing. Note absence of notable effects on this variable following 72 h of tachypacing. (**D**,**E**) VERP evaluated using double threshold stimulation intensity in the Rt. pacing site or the Lt. pacing site. Note that the baseline VERP is significantly longer in the Lt. pacing site. RV tachypacing caused a paradoxal effect by significantly reducing the Lt. VERP and an opposite tendency in the Rt. VERP creating a remarkable difference between the VERP of both sites. Note that such effect did not occur by similar application of BiV pacing.

RV tachypacing for 72 hours significantly prolonged the QTc-NSR relative to baseline, a phenomenon which was not detected following BiV tachypacing (Fig. 3A). Analysis of QT interval during overdrive pacing of each site (100 ms cycle-length) exposed a non-significant tendency for shorter baseline value during left (Lt.) site pacing (Fig. 3B, 71.2 ± 3.1 ms vs. 78.3 ± 2.6 ms; n = 9; p = 0.07). Following 72 h, paced-QT during Lt. site pacing tended to increase in the RV-paced group and decrease in the BiV-paced group, leading to a marked final difference between the two pacing modes in paced QT at this site (Fig. 3B).

To further elucidate the electrophysiological details, we analyzed epicardial activation time and VERP during right (Rt.) site pacing and Lt. site pacing. Epicardial activation was not affected by the different pacing modes (Fig. 3C). However, VERP was markedly affected. At baseline, VERP was slightly but significantly longer in the Lt. site (Fig. 3D; 50.4 ± 0.8 ms 47.3 ± 1.2 ms; n = 12). RV pacing led to notable shortening of the Lt. site VERP compared to both baseline and BiV pacing. In contrast, the RV pacing also led to a non-significant tendency of increase of the Rt. site VERP (p = 0.07). The net effect of both changes was a marked dispersion of the VERP, which was specific to the RV-paced group and was not detected following BiV pacing (Fig. 3E).

### Molecular consequences of sustained RV vs. BiV pacing modes

**Figure 4:**
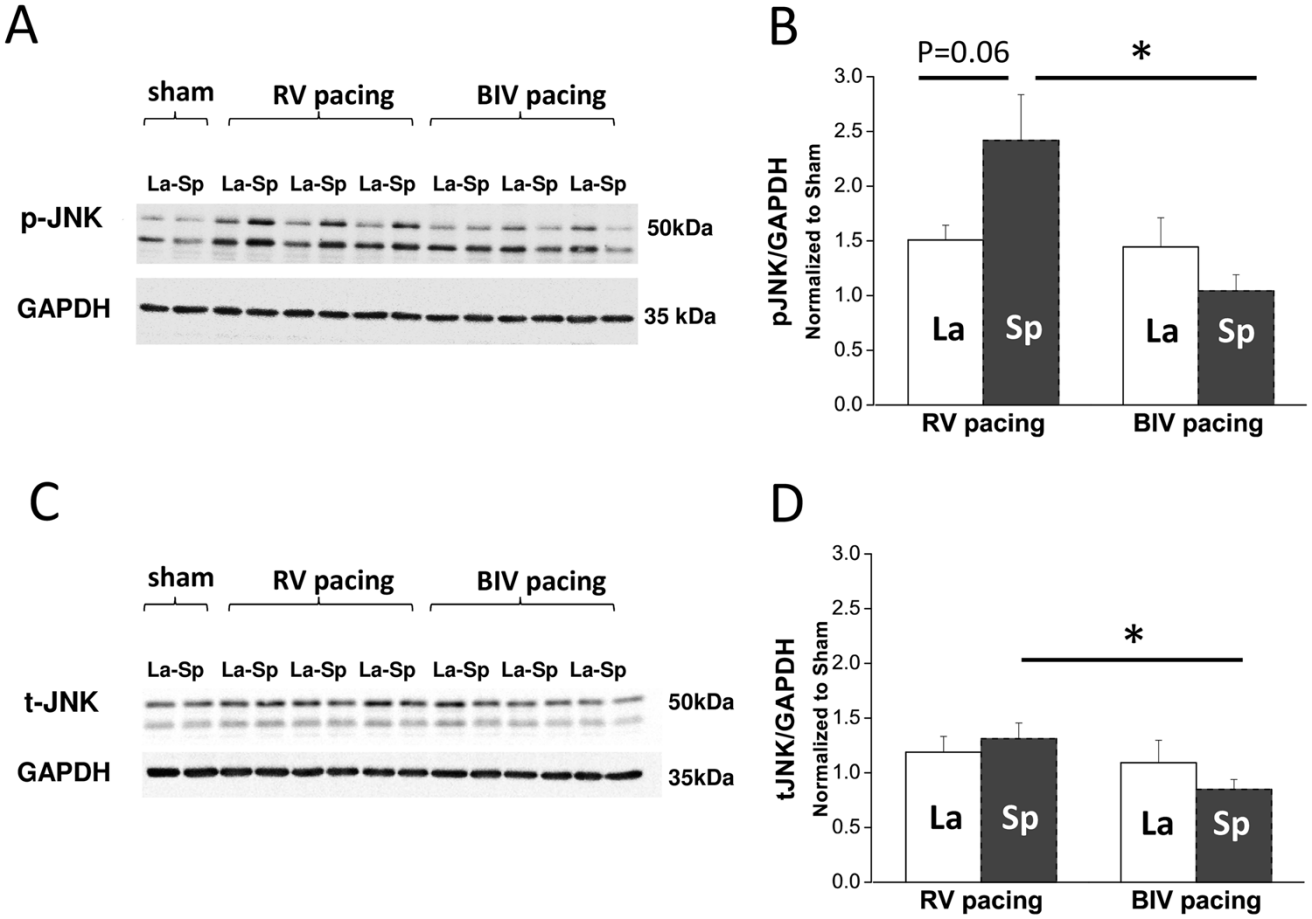
RV tachypacing induces ‘molecular polarization’ of JNK activation between the septum and lateral wall. Biochemical analysis of JNK in rats subjected to either RV tachypacing or BiV tachypacing at a CL of 100 ms for 72 hours. La = lateral wall, Sp = septum. (**A**) Immunoblot demonstrating phospho-JNK (p-JNK) levels relative to GAPDH (loading control). Note the marked increase of p-JNK in the septum of rats exposed to RV tachypacing, which did not occur in response to BiV pacing. Cropped blots are shown. Uncropped blots are presented in Supplementary Fig. 5S. (**B**) Bar graph summarizing the p-JNK densitometry results of 6 individual rats in each pacing mode. (**C**) Immunoblot for total-JNK (t-JNK) relative to GAPDH. Cropped blots are shown. Uncropped blots are presented in Supplementary Fig. 5S. (**D**) Bar graph summarizing the t-JNK densitometry results of 6 individual rats in each pacing mode. Note small but significant effects of RV tachypacing on the total expression of JNK in addition to the more profound effect on phosphorylation levels.

**Figure 5:**
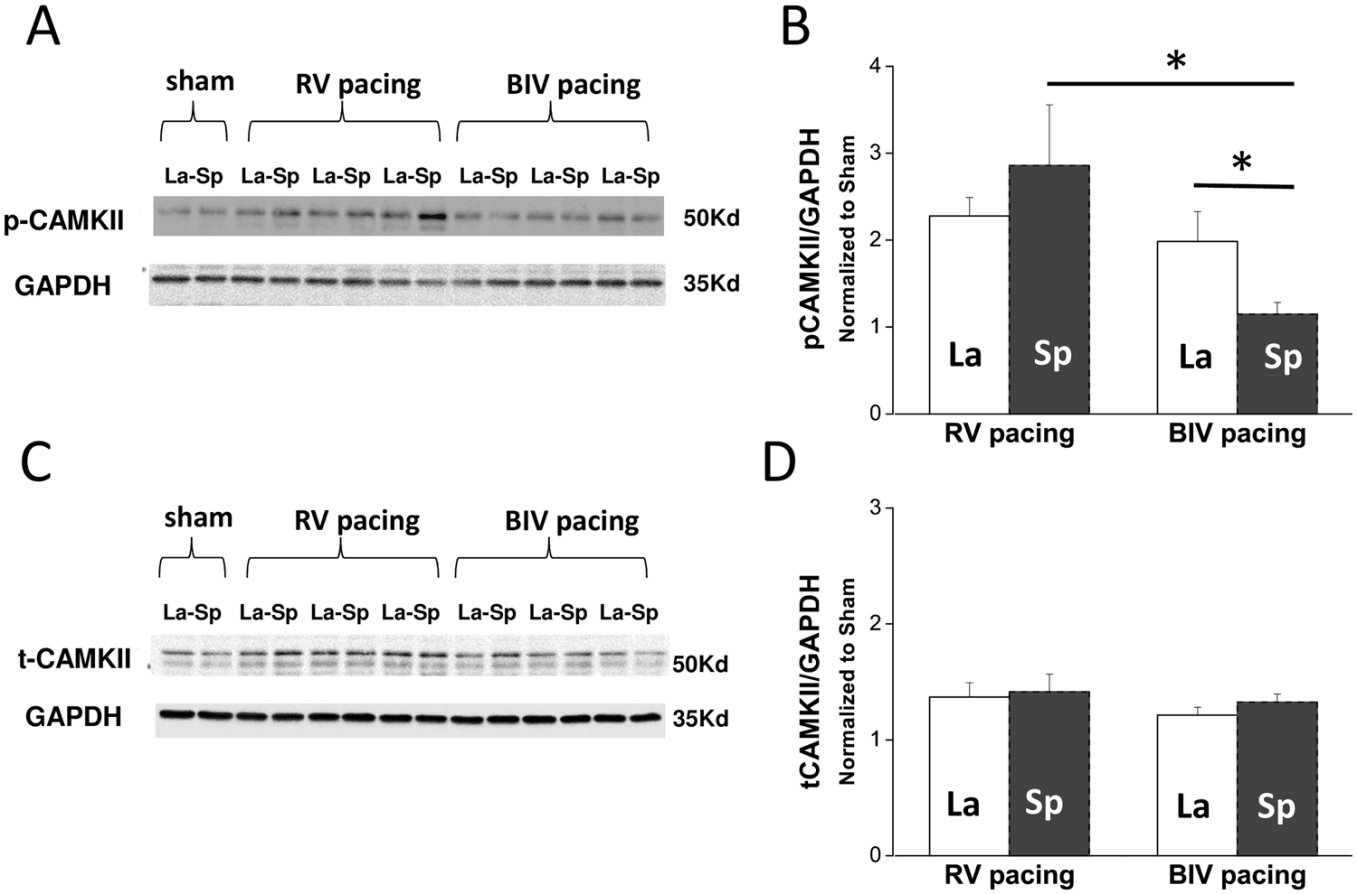
RV tachypacing induces global activation of CAMKII. Biochemical analysis of CAMKII in rats subjected to either RV tachypacing or BiV tachypacing at a CL of 100 ms for 72 hours. La = lateral wall, Sp = septum. (**A**) An immunoblot demonstrating phospho-CAMKII (p-CAMKII) levels and GAPDH (loading control). Cropped blots are shown. Uncropped blots are presented in Supplementary Fig. 5S. (**B**) Bar graph summarizing the p-CAMKII densitometry results of each pacing mode. Note the marked increase of p-CAMKII in both septum and lateral wall of rats exposed to RV tachypacing. In comparison with the RV-paced rats, BiV-paced rats demonstrated reduced p-CAMKII levels mainly in the septum. (**C**) Immunoblot for total CAMKII. Cropped blots are shown. Uncropped blots are presented in Supplementary Fig. 5S. (**D**) Bar graph summarizing the CAMKII densitometry results at each pacing mode.

**Figure 6:**
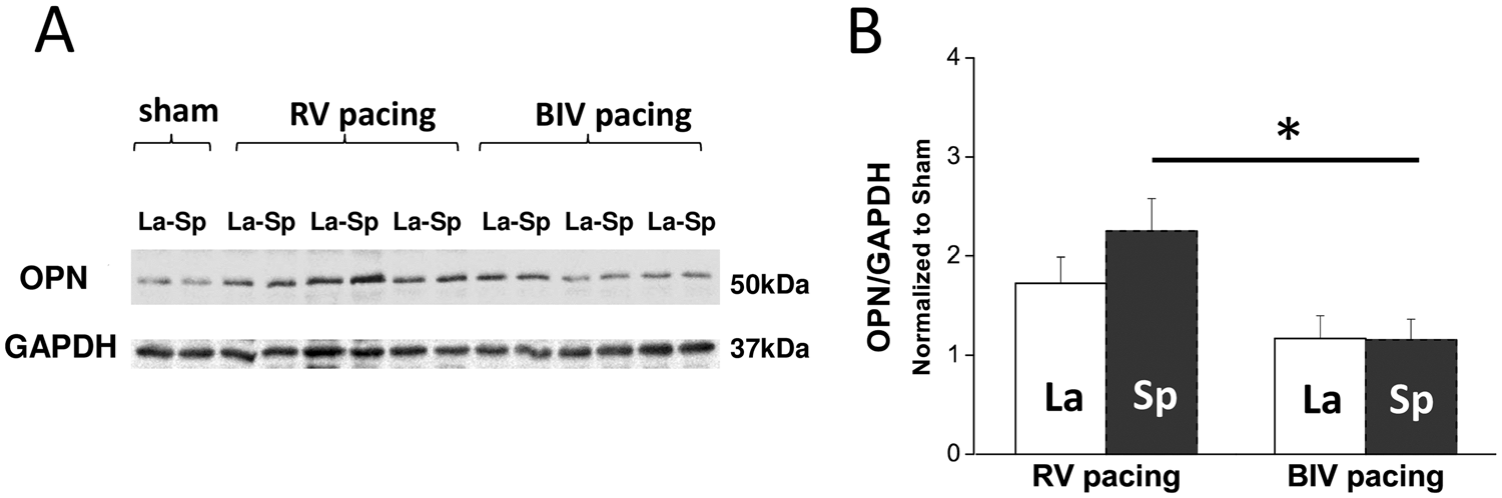
RV tachypacing induces global activation of osteopontin. Biochemical analysis of osteopontin (OPN) levels in rats subjected to either RV tachypacing or BiV tachypacing at a CL of 100 ms for 72 hours. La = lateral wall, Sp = septum. (**A**) Immunoblots demonstrating OPN and GAPDH (loading control). Cropped blots are shown. Uncropped blots are presented in Supplementary Fig. 5S. (**B**) Bar graph summarizing the densitometry results of 6 individual rats in each pacing mode. Note the marked increase of 50 kDa OPN in both septum and free wall of rats exposed to RV tachypacing. In comparison, BIV pacing demonstrated significantly reduced levels in the septum.

To further characterize the differential effects of RV and BiV pacing modes on the myocardial level, samples from the early-activated (septum, Sp) and late-activated (lateral wall, La) parts of the mid-LV were analyzed focusing specifically on molecules and signals that were previously implicated in the pathophysiology of electromechanical dyssynchrony^2, 3, 23^. Analysis of phosphorylated ERK, P38 or AKT did not reveal differential effects between the animals exposed to the different pacing modes (Supplemental Fig. 2S). However, we found that p-JNK was notably elevated regionally in the Sp relative to the La tissue in RV-paced animals (Fig. 4A), a trend which almost reached the cutoff of significance (n = 6, p = 0.06). In addition, in comparison with the BiV-paced group, p-JNK and to a lesser extent also t-JNK were significantly higher in the Sp tissue of the RV-paced animals (Fig. 4B–D). Thus, dyssynchrony appears to induce ‘molecular polarization’ of JNK signaling, which is effectively prevented by BiV pacing. CAMKII signaling was also found to be differentially affected by RV vs. BiV pacing. While p-CAMKII levels of the RV-paced animals appeared to be globally elevated in both Sp and La tissue relative to shams (Fig. 5A). BiV pacing elevated CAMKII phosphorylation only in the La myocardium (Fig. 5A,B). The total CAMKII was not affected by pacing (Fig. 5C,D). A rather similar pattern of increased levels in RV-paced group, which was prevented by BiV pacing was also noted in the expression of osteopontin. This finding reached significance in the Sp tissue (Fig. 6) and was specific to the 50 kDa isoform previously associated with RV failure in rats^34^.

Levels of Cx43 were also evaluated following RV vs. BiV pacing. While expression appeared to be globally increased in the tachypaced animals relative to shams, it did not consistently vary between the different pacing modes (Supplemental Fig. 3S-A,B). Histologically, we also evaluated the effects of the different pacing modes on Cx43 localization. Proper evaluation was not possible in all preparations due to technical limitations. However, in some animals clear data could be obtained from the Sp and La zones. In general, these images may imply higher level of Cx43 in the tachypaced animals (Supplemental Fig. 3S-C). However, the pattern of expression was variable and we could not identify any marked differences between the Cx43 pattern of RV-paced and BiV-paced groups. Finally, RNA levels of genes that were previously suggested to be affected by dyssynchrony or systolic dysfunction were evaluated including caspase 3, cartilage oligomeric matrix protein (Comp), Dickkopf-related protein 3 (DKK3), natriuretic peptide A (Nppa), natriuretic peptide B (Nppb) and secreted phosphoprotein 1. For two of these genes (caspase 3 and NppA) the pattern seemed to be differentially affected by the RV vs. BiV pacing modes (Supplemental Fig. 4). However, rather high variability was noted within the animals of each group and data did not reach the level of significance.

## Discussion

In this study, we further characterized our recently introduced model of pacing-induced dyssynchrony and resynchronization in instrumented rats^25^. This model is based on an implantable device that permanently locates two bipolar-electrodes on the rodent heart. The double-site nature of this methodology is both unique and critical to support systematic and well-controlled comparisons between different pacing modes, in ways which cannot be done using single site devices^23, 35^. Our previous speckle-tracking echocardiography findings revealed that electromechanically, rat cardiac pacing mimics important features seen in the large mammalian heart. However, the actual effects of the RV vs. LV-based pacing modes on cardiac performance were not described yet. Here, we aimed to reach this goal using P-V loop hemodynamic analysis, which is established as the most rigorous and comprehensive way to assess intact heart function^36, 37^. Moreover, the model was also successfully utilized for the first time, to apply persistent pacing in conscious freely moving rats and analyze various effects of prolonged RV vs. BiV pacing. In the next paragraphs, we will discuss the main findings of these two accomplished goals in detail.

### Hemodynamic findings

By instrumenting RA + RV electrodes we were able to dissect between the effects of RV pacing per-se on the rat heart and the additional effect on AV synchrony. Utilizing pacing modes with constant heart rate and fairly equivalent AV synchrony (A vs. A + RV) we found that A + RV pacing did not influence measures of preload (LV end diastolic pressure or volume; see Table 1), but markedly affected LV performance in ways that largely resemble the acute systolic and diastolic impairments induced by electromechanical dyssynchrony in the large mammalian heart^26–29^. An increment in LV ESV without a concomitant rise in LV ESP accordingly indicate a rightward shift of systolic P-V relationship and support deterioration of myocardial contractility during A + RV pacing. The deterioration of LV contractility during A + RV pacing was further supported by the reduction of dP/dt max. In addition, markedly reduced SW consistently shows that the depressed contractility is associated with impairment of LV pump function. Since RV pacing also increases the degree of inhomogeneity in contraction and relaxation^30^, it is not surprising that ventricular pacing also impaired ventricular relaxation parameters. Similarly to the systolic function, we observed lower dP/dt min and prolonged Tau during A + RV pacing, as compared with A pacing, indicating that pacing induced LBBB pattern slows the process of isovolumic relaxation and impairs the diastolic function of the LV. Overall this analysis clearly supports the notion that the hemodynamic effects of RV pacing per-se largely resemble the effects of LBBB and RV pacing in the large mammalian heart.

A prominent increase of Ea, which reflects the net arterial load that is imposed on the LV^31^, is an important additional consequence of rodent RV pacing that we revealed. Ea is a modulator of cardiac performance^38^ and a potent predictor of cardiovascular outcomes^39, 40^. Elevated Ea increases myocardial energetic costs to eject a given amount of blood^41, 42^. In addition, Ea is also a modulator of diastolic processes, and arterial load can prolong relaxation in humans and animals^43–45^. Previous work indicated that RV pacing in individuals with normal LV function is associated with a 65% increase in cardiac norepinephrine spillover^46^. Thus, it is highly plausible that the elevated Ea we observed here reflects reactive increase in cardiac norepinephrine spillover due to the reduced cardiac performance during RV pacing. Interestingly however, increased Ea is not typically observed in humans exposed to RV pacing^32^. This discrepancy might reflect differences in the intensity of pacing-induced norepinephrine spillover or vasomotor response between rodents and humans. In addition, in humans, RV pacing may induce mitral regurgitation, which can actually reduce the load that is imposed on the LV and thus decrease Ea^33^. In the rats, our echocardiographic analysis did not reveal indications for such pacing-induced mitral regurgitation. Of note, increased sympathetic activity as a result of reduced cardiac performance can lead to electrophysiological changes and ventricular arrhythmias due to the dispersion of ventricular repolarization^47^. Such dispersion of ventricular repolarization is in agreement with the VERP changes that we noted in the rats following prolonged RV tachypacing in the present study (Fig. 3).

By further comparing between different ventricular pacing modes (Table 2), we found that LV systolic function indices were markedly improved during LV-based pacing modes compared with RV pacing. In contrast, indices related to LV diastolic function did not differ significantly between the three pacing types. Since LV EDV was significantly higher while LV EDP remained the same during LV-based pacing modes, it appears that the external constraint to LV filling by the RV through the interventricular septum was ameliorated compared with RV pacing. Such relief of an external constraint can indeed induce an increase in EDV despite a similar EDP, thereby increasing SV and SW by the Starling mechanism^48^. A probable mechanism for a reduction in external constraint may be related to induction of a phase shift in the timing of LV filling relative to RV filling, resulting in the timing of events being moved forward in the LV. This effectively results in LV filling occurring at a time when RV pressure and volume are low; hence, RV diastolic pressure is likely to be lower at any given LV diastolic volume^48^. The Ea reduction during LV-based pacing modes compared with RV pacing is consistent with compensatory changes of arterial resistance in response to the enhanced cardiac performance during the LV-based pacing modes. In accordance with previous findings in humans, we may postulate that increased LV performance during the LV-based pacing modes can augment activity of vagally innervated receptors located in the left ventricle to tonically inhibit the vasomotor center^49^.

Inferior vena cava occlusion attempts, which were performed in order to dissect the various contributors to the prominent difference in cardiac performance between RV pacing and LV-based pacing modes were technically challenging and did not yield consistent and conclusive results at our hands. Thus, while we can speculate that a combination of reasons including increased systolic function, better arterial load and augmented LV filling all contributed to the marked net differences in cardiac performance between RV-pacing and LV-based pacing modes, at present we could not determine the actual contribution of each of the mentioned above factors.

### Myocardial properties following persistent RV vs. BiV tachypacing

A major goal in developing the current animal model is to allow studies of the complex molecular and cellular biology of cardiac tissue in response to electromechanical dyssynchrony and resynchronization. In this regard, our current ability to directly compare between the effects of persistent RV tachypacing and BiV tachypacing applied for three days in conscious freely moving rats, is an important achievement.

In terms of electrophysiology, the QT prolongation in response to RV tachypacing but not BiV tachypacing, is a strong indication that the two modes of pacing indeed affect myocardial properties differentially in the rat. However, while this finding itself might only reflect the different hemodynamic status imposed by the two pacing modes, VERP measurements clearly demonstrated differential effects of RV tachypacing on the right and left ventricles, including shortening of VERP in the LV site and a reverse tendency in the RV site. Such findings support an inhomogeneous effect of RV pacing on the action potential duration (APD) of the rat heart. Moreover, considering the unchanged epicardial activation time in both groups, the observed differences in paced-QT during LV site pacing also support prolongation of APD in the RV free wall. Although our current analysis cannot determine the VERP in the early-activated septal part of the LV, we may speculate that it is also prolonged and contributes to the global increase in non-paced QT following RV tachypacing. Regional changes of VERP in the RV paced rats are somewhat reminiscent to published data in the dog model of LBBB where such changes were related to regional differences in load as well as to the extent of myocardial stretch applied at different time points during the action potential^50^. In rabbits, myocardial stretch applied during phase 2 of the action potential shortens its duration; conversely, when applied during phase 3 or 4 of the action potential, its duration is prolonged^51^. This pathophysiology is indeed what one can expect during dyssynchrony, where the lateral free wall undergoes early systolic stretch, approximately during phase 2 of the action potential, while the septum undergoes late systolic stretch, most likely during phase 3–4 of the action potential. Whether similar effects of mechano-electrical feedback also apply to the rat heart, is a possibility that will have to be further examined to understand the underlying mechanisms of our current electrophysiological findings. In addition, inhomogeneous regional electrophysiological effects following RV tachypacing might increase the risk for malignant ventricular arrhythmias^52^ and such prediction will be important to study further using the current model.

Our biochemical results comparing myocardial tissue from the septal wall and the lateral wall of the LV, also support differential effects of RV pacing on the early-activated and late-activated regions as previously suggested in the mouse heart^23^. In contrast to Bilchick *et al*.^23^, here we could directly compare between RV-paced tissue and BiV-paced tissue, under similar beating rate and similar AV synchrony, a mission which is impossible using single site pacing methodology. The most important and somewhat unexpected finding here was a differential effect of RV tachypacing on JNK signaling, in contrast with P38, ERK and AKT previously implicated in the myocardial response to dyssynchrony in the dog heart^2, 3^. It will be important to further explore whether these discrepancies reflect species dependent differences, or alternatively, it might reflect temporal variations due to the relatively short period of pacing we used here in comparison with the 3–6 weeks period applied in the dog model^2, 3^. In any case, considering the fact that JNK is a stress-activated protein kinase with clear importance in the myocardium^53, 54^, this new finding and its implications on the pathophysiology of myocardial dyssynchrony should be further explored in details. It is for example known that JNK is a major regulator of cardiac KChIP2 gene expression, and activation of JNK can diminish the transient outward K+ current (Ito) by transcriptional down-regulation of KChIP2^55^. Whether such mechanism may contribute to differential changes in APD in the RV-paced rat heart is an important possibility that might be explored based on the current findings.

Other observed changes in the RV-paced rats including CAMKII activation and increased expression of osteopontin can reflect the profound effects of this dyssynchronous mode of tachypacing on the rat myocardium as opposed to BiV tachypacing. Thus, we may expect that with further prolongation of the tachypacing period profound differences in cardiac function would be noted between animals exposed to RV tachypacing and those exposed to BiV tachypacing. This may lay the foundation for a rat model, which is rather similar to the dog model of dyssynchronous heart failure induced by tachypacing in combination with LBBB^2, 3^. Moreover, by using rats it should be rather straightforward to combine our model with other insults leading to myocardial dysfunction such as hypertension, diabetes mellitus, high output failure and more. Further echocardiography regional dyssynchrony assessment in parallel with ventricular arrhythmias substrate evaluation^56^, is another important future direction. In summary, our current findings support the notion that the instrumented rat model is an important new advance in this field of research and may be used to shed light on fundamental effects of dyssynchrony and resynchronization on the tissue level.

## Materials and Methods

The study was carried out in strict accordance with the Guide for the Care and Use of Laboratory Animals of the National Institute of Health. All the experiments reported in this study were approved by the institutional ethics committee of Ben-Gurion University of the Negev, Israel (IL-38-06-2013, IL-84-12-2015). Adult male Sprague-Dawley rats were obtained from Harlan Laboratories (Rehovot, Israel). The animals were kept under standardized conditions throughout the study, according to home office guidelines: 12:12 light:dark cycles at 20–24 °C and 30–70% relative humidity. Animals were free-fed autoclaved rodent chow and had free access to reverse osmosis filtered water. At the end of the experiments animals were sacrificed by extraction of the heart under deep Nembutal-induced anesthesia.

### Surgical implantation of the rat pacing device

The implantable device and the surgical technique that were utilized to permanently locate two bipolar-electrodes in predetermined epicardial locations were previously described in detail^25, 57^. Briefly, the electrical connections to the epicardial surface were done using miniature-bipolar hook electrodes that were designed by our group for various *in-vivo* electrophysiological applications in rodents^25, 57, 58^. Each electrode contains a distal head with two sharp tungsten pins that are curved and isolated by insulted coating up to their tips. By means of small lateral thoracotomy the electrode can be fixed on the selected epicardial surface without the need for additional suturing. The implantable device is composed of an 8 pin ‘female’ connector that is attached by highly flexible insulted electrical wires (AS155–36, Conner Wire, Chatsworth, CA) to a set of two miniature-bipolar hook electrodes. Additional electrodes for peripheral pseudo-ECG measurements are also implanted subcutaneously. Electron beam radiation is applied for sterilization of the device before its use. For device implantation rats (250–320 g) were anesthetized (ketamine/xylazine 75/5 mg/kg, IP) and mechanically ventilated. The animals were placed on a warmed heating pad and under sterile conditions the two miniature-bipolar hook electrodes were implanted on the desired sites. Following chest closure the 8-pin ‘female’ connector was exteriorized through the skin of the back. Postoperative recovery and analgesia were done as described previously^25^. Buprenorphine (0.1 mg/Kg) was given by subcutaneous injection during immediate recovery and every 24 hours during the first 3 days. In addition, Dipyrone (400 mg/500 ml) was added to the drinking water during the first 3 days. The animals were monitored on a daily basis for signs of stress or inappropriate weight loss, according to guidance from the Ben-Gurion University veterinary services (assured by the Office of Laboratory Animal Welfare, USA (OWLA) #A5060-01, and fully accredited by the Association for Assessment and Accreditation of Laboratory Animal Care International (AAALAC)).

### Study design

Overall 39 successfully implanted rats were evaluated in this work. The study contained two principle parts: 1. Acute hemodynamic measurements during various pacing modes (n = 23). In this section each animal served as its own control and the effects of various pacing modes were compared in each animal as previously done for strain analysis^25^. 2. Comparison between the effect of sustained RV tachypacing and BiV tachypacing (600 BPM) applied for 72 hours (n = 14). In section #1 hemodynamic measurements were initially done in animals implanted with right atria (RA) and RV electrodes (group A) to fully characterize the effect of RV pacing with and without optimal atrio-ventricular (A-V) coupling (n = 8). Next, electrodes were implanted on the RV and LV (group B) to directly compare between the different ventricular pacing modes using conventional P-V loop recordings (n = 8), inferior vena-cava occlusion studies (n = 5, excluding 6 additional with technical difficulties) and echocardiographic evaluation directed towards mitral regurgitation and atrial dimensions assessment during the different pacing modes (n = 5). In part #2 animals were divided into three groups: shams, which were connected to the system without pacing (n = 2), sustained RV pacing (n = 6) and sustained BiV pacing (n = 6).

### P-V loop recordings during various pacing modes

Rats were evaluated 6–8 days after implantation of the pacing device. Animals were anesthetized by 3% isoflurane in an induction chamber followed by constant administration of 2% isoflurane with supplemental O_2_ through a face mask. Body temperature was rectally monitored and maintained at 37 ± 0.5 °C by a circulating water warming pad. Electrical stimulation (2 ms square pulses) was applied using isolation units (Iso-Flex, AMPI, Israel) at a double diastolic threshold. A Labview 7.1 based program previously developed in our laboratory^57^ was used to control electrical stimulation as well as ECG recordings (RMC1100, Nihon Kohden, Foothill Ranch, CA) through an A/D converter (PCI-6024E, National Instruments, Austin, TX) as previously described^25^.

In each animal the right common carotid artery was exposed and a 1.9-Fr pressure-volume (P-V) catheter (FTH-1912B-8018, Transonic Systems Inc, Ithaca, NY) was inserted into the right common carotid artery and passed in a retrograde fashion through the aortic valve into the LV to monitor pressure and volume as well as admittance magnitude and phase angle signals in the LV. Data were acquired using a ADI LabChart acquisition and analysis software which was also used for offline analysis. Signals were acquired at a sampling rate of 1,000 Hz using an Advantage PV system (model ADV500, Transonic Systems Inc). The steps involved in the setup and use of the Advantage system, i.e., catheter calibration and measures of myocardial conductivity and permittivity were done as previously described in the literature^59–61^. Based on previous echocardiographic measurements from our laboratory under isoflurane anesthesia we estimated stroke volume of 0.9 μl/g of body weight for each animal. While this estimation might be somewhat biased from the absolute values, our analysis focused on the comparison of the effects of different pacing modes in each animal. Thus, the relative changes between the pacing modes were the main focus of our study. Following equilibration, pressure–volume curves were acquired during various pacing modes using continuous override pacing at a 150 ms cycle length (CL). The P-V loop acquisition protocol was as follows: Following optimal positioning of the catheter in the LV, it was fixed in place and LV P-V loops (1–2 minutes each) were sequentially recorded in sinus rhythm followed by override pacing at the different pacing sites. In group A the modes of the pacing were: (1) RA pacing alone; (2) sequential RA-RV pacing at the longest AV interval producing a right ventricular activation sequence without fusion as confirmed by QRS morphology (Fig. 1A); (3) RV pacing alone. In-group B the modes of the pacing were: (1) RV pacing alone; (2) LV pacing alone; (3) simultaneous BiV pacing. Analyzed hemodynamic parameters of the LV included: mean pressure (Pmean), developed pressure (Pdev), end-systolic pressure (ESP), end-diastolic pressure (EDP), end-systolic volume (ESV), end-diastolic volume (EDV), stroke volume (SV), arterial elastance (*E*_a_), ejection fraction (EF), stroke work (SW), the maximal slope of the systolic pressure increment (dP/d*t*_max_), the diastolic pressure decrement (dP/d*t*_min_), and the time constant of isovolumic relaxation (Tau). At the end of all P-V loop recordings animals were sacrificed and correct positioning of the epicardial electrodes was confirmed by post-mortem operation. For description of inferior vena-cava occlusion attempts under RV vs. BiV pacing see supplemental methods.

### Echocardiography

Analysis was done under isoflurane sedation (1%) as previously described^25^ with a Vivid 7 echocardiography system (GE Healthcare, Milwaukee WI) and 10S probe. Following optimal positioning of the transducer in an apical four chamber view, it was fixed in place by the operator, while clips were sequentially recorded during override pacing (150 ms Cycle length) under the different pacing modes (no pacing, RV, LV and BiV). Presence or absence of mitral regurgitant jet was assessed with 2D color Doppler and continuous-wave Doppler. Maximal atrial diameter in the apical four chamber view was also evaluated during the different pacing modes.

### Long term pacing and effective refractory period measurements in implanted rats

On the 6th postoperative day animals were placed in dedicated pacing and recording cages where the back connector was linked by an elastic cable to the pacing and recording apparatus as previously described^57^. Pacing was done using optically isolated units (STG4002-16mA, Multichannels, Reutlingen, Germany). Pseudo ECG and epicardial signals were recorded using conventional amplifier (Model 1700, A-M Systems, Carlsborg, WA). The animals were allowed to adapt for 24 h in the cage. On the 7th postoperative day, baseline pseudo ECG recordings were recorded and measurements of ventricular effective refractory period (VERP) were evaluated through each pacing electrode (RV and LV). VERP was determined at twice-diastolic threshold as the longest S1-S2 interval that failed to generate a response. The programed stimulation protocol included ten S1 stimuli at 100-ms cycle length, followed by S2 decremented in 2-ms intervals. QT and epicardial activation time measurements were done as previously described^25^. QTc was calculated using normalized Bazett’s equation adapted for rats: QTc_n_ − B = QT/(RR/f)^1/2^, where f is the normalization factor according to the basal RR duration (150 ms for rats)^62^.

After baseline measurements, continuous RV pacing (n = 6) or BiV pacing (n = 6) was initiated at a 100 ms CL. Confirmation of effective pacing was continuously monitored through online pseudo ECG recordings. For the BiV pacing group we routinely confirmed effective pacing through each of the pacing channels (RV and LV) at least twice daily. At the end of 72 hours of pacing all electrophysiological parameters were re-evaluated, before the animals were sacrificed.

### Biochemical and histological analysis

All animals exposed to 72 hours of RV pacing (n = 6), BiV pacing (n = 6) or sham-operation with no pacing (n = 2) were sacrificed and tissue samples from the septum (early activated) and the free wall (late activated) of the LV were snap frozen in liquid nitrogen. Protein lysates were obtained in RIPA buffer containing protease and phosphatase inhibitors and prepared for immunoblot analysis as previously described^63^. Membranes were incubated with primary antibodies including: rabbit anti-phospho-ERK 1/2 (SAB1306604; Sigma-Aldrich); rabbit anti-phospho-p38 (4511; Cell Signaling Technology); rabbit anti-phospho-JNK (4668; Cell Signaling Technology); mouse anti-total-JNK (sc-7345; Santa Cruz), rabbit anti-phospho-CaMKII (12716; Cell Signaling Technology); rabbit anti-total-CaMKII (4436; Cell Signaling Technology), rabbit anti-phospho-Akt Ser473 (4060; Cell Signaling Technology); mouse anti-osteopontin (sc-21742; Santa Cruz Biotechnology); rabbit anti-Connexin 43 (3512; Cell Signaling Technology). GAPDH was evaluated using rabbit anti-GAPDH (sc-25778; Santa Cruz Biotechnology) and served as loading control. Filters were extensively washed in TBS-T and then were incubated with horseradish peroxidase-conjugated secondary antibody. Secondary antibody was a species-specific horseradish peroxidase-conjugated goat anti-rabbit (sc-2004; Santa Cruz Biotechnology) or goat anti-mouse (sc-2004; Santa Cruz Biotechnology). Bands were visualized by using the ECL system (MicroChemi 4.2, DNR Bio-Imaging Systems Ltd) and were quantified with computerized densitometry and ImageJ (version 1.24) software. Densitometric values were reported after normalization for gel-loading differences and relative to the average value obtained from protein extracts of sham-operated control rats. For description of real-time QT-PCR analysis and histological analysis of connexin43 see supplemental methods.

### Statistical analysis

Values are expressed as means ± SE. In part #1 comparison between the different pacing modes was done utilizing 1-way ANOVA of repeated measures and Tukey multiple comparisons post-test. Inferior vena-cava occlusion analysis data was compared using Wilcoxon rank test. In part #2 comparisons were done using paired or unpaired t-test as required. For n of less than 6 or parameters that are not normally distributed non-parametric testing was performed. The criterion for significance was set at *P* < 0.05. Unless otherwise stated p-values are displayed graphically as follows: *p < 0.05, **p < 0.01, ns = not significant.

## Electronic supplementary material

Supplementary data
