# Supplementary material for: Prominent differences in left ventricular performance and myocardial properties between right ventricular and left ventricular-based pacing modes in rats
Source: Sci Rep. 2017 Jul 19;7:5931. doi: 10.1038/s41598-017-06197-w (PMC5517524; doi:10.1038/s41598-017-06197-w)
Supplement: Supplementary file 1 — Supplementary data [file 41598_2017_6197_MOESM1_ESM.docx]

**SUPPLEMENTAL MATERIAL**

**Prominent differences in left ventricular performance and myocardial properties between right ventricular and left ventricular-based pacing modes in rats**

**Authors:** Wesam Mulla^1^, Sharon Etzion^2^, Sigal Elyagon^1^, Roni Gillis^1^, Michael Murninkas^1^, Yuval Konstantino^3^, Ingra Mannhardt^4^, Thomas Eschenhagen^4^, Noah Liel-Cohen^3^ and Yoram Etzion^1,2^

**Affiliations:**

**^1^**Cardiac Arrhythmia Research Laboratory, Department of Physiology and Cell Biology, Faculty of Health Sciences, Ben-Gurion University of the Negev, Beer-Sheva, Israel.

**^2^**Regenerative Medicine & Stem Cell Research Center, Ben-Gurion University of the Negev, Beer-Sheva, Israel.

**^3^**Cardiology Department, Soroka University Medical Center, Beer-Sheva, Israel.

**^4^**Department of Experimental Pharmacology and Toxicology, Cardiovascular Research Center, University Medical Center Hamburg-Eppendorf, Hamburg, and DZHK (German Center for Cardiovascular Research), partner site Hamburg/Kiel/Lübeck, Hamburg, Germany.

**Address for Correspondence:** Yoram Etzion M.D., Ph.D.

Cardiac Arrhythmia Research Laboratory, Department of Physiology and Cell Biology, Faculty of Health Sciences, Ben-Gurion University of the Negev. P.O. Box 653, Beer-Sheva 84105, Israel

Tel: +972-8-647-9901, Fax: +972-8-647-9875

e-mail: [tzion@bgu.ac.il](mailto:tzion@bgu.ac.il)

**Supplemental Methods**

Inferior vena-cava occlusion under RV vs. BIV pacing

To gain insights into the effects of RV and BiV pacing on load independent parameters ^1,2^, inferior vena-cava occlusion was performed by transiently compressing the inferior vena cava under a diaphragm with a cotton-tipped applicator. Initially, several attempts were made under spontaneous respiration. However, we suffered great difficulty in analyzing these results due to respiration artifacts and only one such attempt appeared informative (Supplemental Figure 1S-A). To overcome this difficulty a set of additional experiments was done under isoflurane anesthesia following intubation and weight-adjusted mechanical ventilation (Inspira; Harvard Apparatus), as well as intravenous application of vecuronium bromide (1 mg kg^−1^) immediately before each vena-cava occlusion attempt. In addition, ventilator was briefly switched off during the occlusion. Under these conditions 4 attempts yielded informative results (Supplemental Figure 1S-B, Supplemental Table-II). Analyzed parameters included: The slope [end-systolic elastance (E_es_)] of the LV end-systolic P-V relationship [ESPVR; according to the parabolic curvilinear model ^3^], preload recruitable SW (PRSW), and the slope of the dP/dt_max_-end-diastolic volume relationship (dP/dt_max_-EDV) were calculated as load-independent indexes of LV contractility. The slope of the LV end-diastolic P-V relationship (EDPVR) was calculated as a reliable index of LV stiffness. Ventriculoarterial coupling was described by the quotient of E_a_ and E_es_ according to Sunagawa and associates ^4^. Cardiac efficiency was calculated as the ratio of SW and P-V area (PVA).

Histological analysis of Cx43

The heart of each paced rat was dissected by short-axis cut and its apical third was embedded in OCT and snap-frozen in liquid nitrogen. Samples were stored at -80 °C. Cryosections (10 µm; Leica CM 3050S) were performed in a transversal short-axis view visualizing left and right ventricle. Tissues were stained automatically (Ventana Benchmark XT) with pre-treatment in CC1 buffer (Ventana Medical Systems; 60 min). Anti-connexin 43 (BD Biosciences, BD610061, 1:100, 30 min, 37 °C) staining was followed by the second antibody from the Ultra View Universal DAB Detection Kit (Ventana Medical Systems; #05269806001) and counter staining with hematoxylin (Ventana Medical Systems; #05266726001, 4 min) and bluing reagent (Ventana Medical Systems; #05266769001, 4 min). Slides were examined and images taken with Zeiss Axioskop 2 and Axiovision software.

Real-time quantitative RT-PCR

RNA samples were extracted from the septum (Sp) and the free wall (La) using Direct-zol™ RNA MiniPrep (Zymo Research, CA, USA). The RNA samples were analyzed for concentration and purity using the NanoDrop ND-100 Spectrophotometer (NanoDrop Technologies, Wilmington, DE, USA). All RNAs displayed a 260/280 optical density ratio >1.9. Synthesis of cDNA for real-time quantitative RT-PCR (q-PCR) was performed usingqScript^TM^ cDNA Synthesis Kit (Quanta BioSciences) according to the manufacture's protocol. Analysis was done using reverse transcriptase-quantitative polymerase chain reaction (RT-qPCR) experiments (Real Time PCR System Instrument – 7300, Applied Biosystems) with PerfeCTa SYBR Green FastMix (Quanta BioSciences). Primers for target genes were designed and synthesized by Agentek, Israel (Supplementary Table I). Cycling conditions were: 95^o^C for 3 min, followed by 40 cycles of 95^o^C for 10 s and 60^o^C for 45 s, and a final melting step (78–99oC) for dissociation. The calculation of relative change in mRNA was performed with the efficiency 2^-ΔΔCT^ method, with the expression of the genes of interest normalized to Hypoxanthine Phosphoribosyltransferase 1 (HPRT1) gene.

**Supplemental Table-1S:** Rat primers for genes of interest.

| **Reverse** | **Forward** |  |  |
| --- | --- | --- | --- |
| CATTGCGAGCTGACATTCCA | GCAGTTACAAAATGGATTACCCTGA | NM_012922.2 | **Casp3** |
| GATTTGGCTGTTATCTTCGGTACC | GGGTAGGATTGACAGGATTGGA | NM_012612.2 | **Nppa** |
| ACCGTCACGCAGTTGTCCTT | CGACACAGACCTGGACGGTT | NM_012834.1 | **Comp** |
| ACAGGGAACAGCAGGCCTCT | CCAAAGGTAGCAATGGGACC | NM_138519.2 | **DKK3** |
| AAACAACCTCAGCCCGTCAC | CGAAATTCCAAGATGGCACATAG | NM_031545.1 | **Nppb** |
| GGTCAGGCTTCAGCCAAGTG | CTCAGAGGAGAAGGCGCATT | NM_012881.2 | **Spp1** |

**Supplemental Figure 1S: Individual variability in the effect of RV pacing during transient inferior vena cava occlusion.** Rats were implanted with RV bipolar electrode and LV bipolar electrode. **A:** Example of PV-loop recordings during RV pacing and BiV pacing. Note a Less steep end-systolic P-V relationship (ESPVR) during RV pacing. **B:** Example of PV-loop recordings during RV pacing and BiV pacing from different rats. Note in this case a steeper end-systolic P-V relationship (ESPVR) during RV pacing.

**Supplemental Table-2S: Transient inferior vena cava occlusion parameters in rats (n=5) subjected to RV pacing vs. BiV pacing**

| Parameter | RV pacing | BIV pacing | P value |
| --- | --- | --- | --- |
| **Ees** (mm Hg/mL) | 1.98±0.86 | 1.90±0.31 | ns |
| **PRSW** (mmHg) | 59.0±13.1 | 82.4±15.8 | ns |
| **dP/dt max-EDV** (mmHg/s/uL) | 14.33±2.65 | 13.83±1.60 | ns |
| **P-V area** (mm Hg*mL) | 15371±3077 | 14607±1582 | ns |
| **Cardiac efficiency** (%) | 68±9 | 80±2 | ns |
| **VAC** | 0.68±0.34 | 0.28±0.02 | ns |
| **EDPVR** (mm Hg/mL) | 0.0089±0.003 | 0.0058±0.001 | ns |

Statistical analysis was performed using Wilcoxon signed-rank test.

**Ees**= end-systolic elastance**, PRSW**= preload recruitable stroke work**, dP/dt max-EDV**= dP/dt_max_-end-diastolic volume relationship**, VAC**= Ventriculoarterial coupling**, EDPVR**= end-diastolic P-V relationship.

**
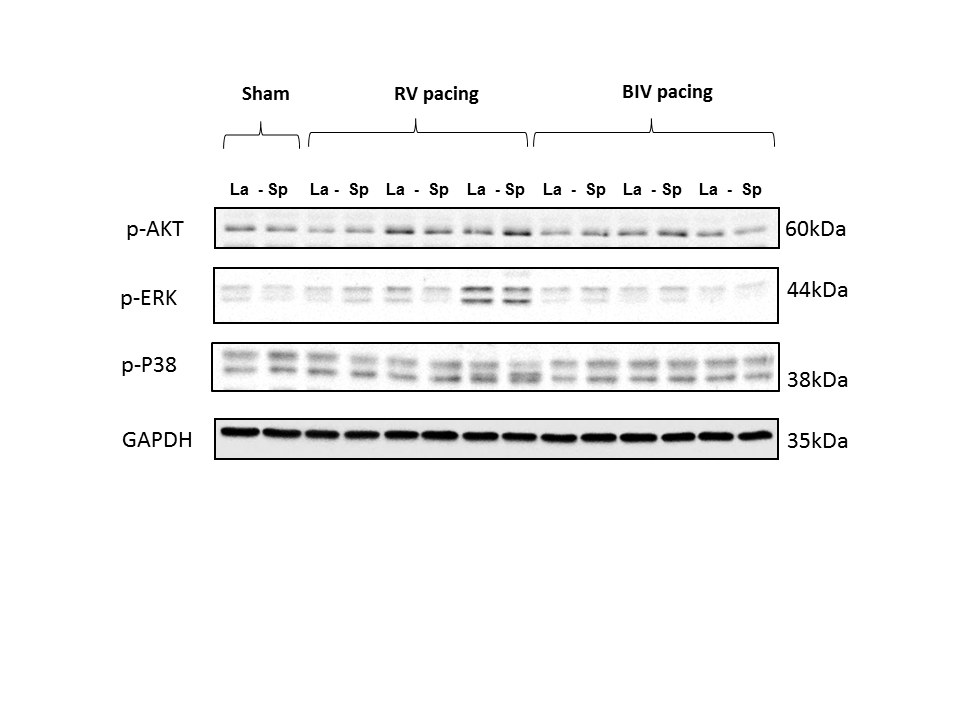
**

**Supplemental Figure 2S: Expressional levels of phosphorylated kinases implicated in dyssynchrony and resynchronization in dogs^5^.** Immunoblots for p-AKT, p-P38 and p-ERK in the septum (Sp) and the lateral wall (La) of rats subjected to either RV tachypacing or BIV tachypacing at a CL of 100 ms for 72 hours. Although some variability was noted, densitometry (not shown) did not reveal any significant differences between RV and BiV paced animals (n=6, for each condition)**.**

**Supplemental Figure 3S: Expressional levels of Cx43 following RV vs. BiV pacing.** Expression of Cx43 in the septum (Sp) and the lateral wall (La) was evaluated in rats subjected to either RV tachypacing or BIV tachypacing at a CL of 100 ms for 72 hours. **A:** Immunoblot demonstrating Cx43 levels and loading controls (GAPDH). **B:** Bar graph summarizing the densitometry results of 6 individual rats in each pacing mode. Statistical analysis did not reveal differences in Cx43 levels between RV and BIV paced rats in both LV regions. **C:** Examples of immuno­staining for Cx43 following the two pacing modes (scale bar 100 μm). Expression pattern was somewhat variable, but did not show clear differences between the two pacing modes in the different LV regions.

**Supplemental Figure 4S: Myocardial gene expression analysis.** In rats subjected to either RV tachypacing or BiV tachypacing at a CL of 100 ms for 72 hours, we analyzed RNA levels of genes that were previously suggested to be affected by dyssynchrony or systolic dysfunction including Caspase 3, Cartilage Oligomeric Matrix Protein (Comp), Dickkopf-related protein 3 (DKK3), Natriuretic Peptide A (Nppa), Natriuretic Peptide B (Nppb) and secreted phosphoprotein 1 (Spp1). La=lateral wall, Sp= septum. Δ indicates the fold change difference between La and Sp regions for each pacing mode. Although some findings suggested increased Δ in the RV-paced group none of the observed changes reached the cutoff for significance.

**
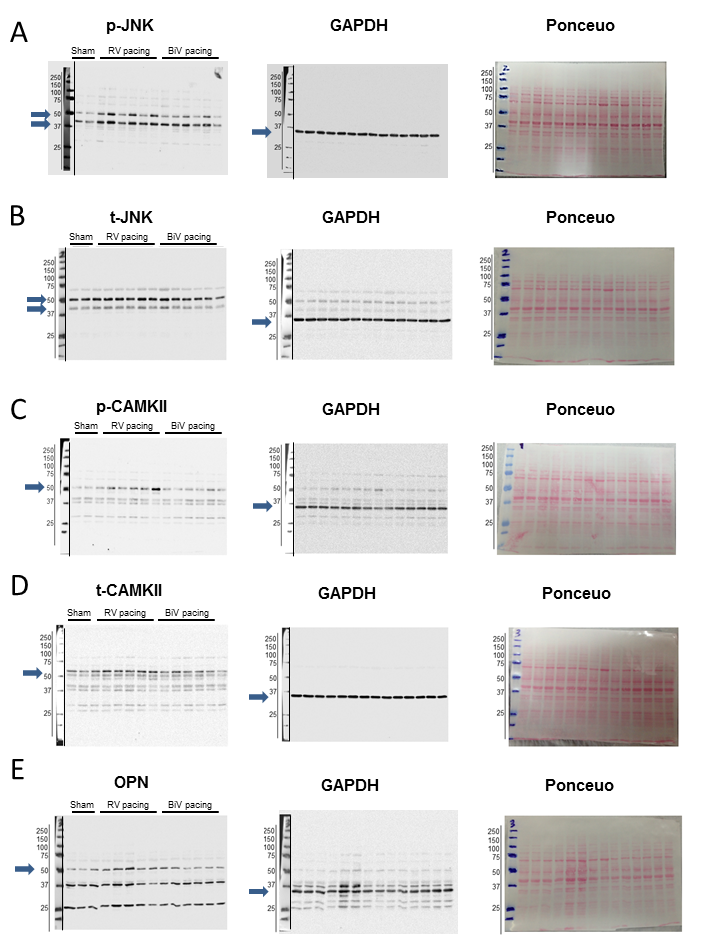
**

**Supplemental Figure 5S: Uncropped gels.** Left column; blots with antibodies of interest. Middle column; GAPDH (loading control) obtained from the same gels following western blotting with the antibody of interest. Right column; Ponceuo staining of the gels. **A-B.** p-JNK and t-JNK, see figure 4 for details. **C-D.** p-CAMKII and t-CAMKII, see figure 5 for details. **E.** osteopontin, see figure 6 for details. Arrows indicate the bands shown in the cropped gels in the paper figures.

**Supplemental references**

1 Pacher, P., Nagayama, T., Mukhopadhyay, P., Batkai, S. & Kass, D. A. Measurement of cardiac function using pressure-volume conductance catheter technique in mice and rats. *Nat Protoc* **3**, 1422-1434, doi:10.1038/nprot.2008.138 (2008).

2 Cingolani, O. H. & Kass, D. A. Pressure-volume relation analysis of mouse ventricular function. *American journal of physiology. Heart and circulatory physiology* **301**, H2198-2206, doi:10.1152/ajpheart.00781.2011 (2011).

3 Kass, D. A. *et al.* Influence of contractile state on curvilinearity of in situ end-systolic pressure-volume relations. *Circulation* **79**, 167-178 (1989).

4 Sunagawa, K., Maughan, W. L., Burkhoff, D. & Sagawa, K. Left ventricular interaction with arterial load studied in isolated canine ventricle. *The American journal of physiology* **245**, H773-780 (1983).

5 Cho, H., Barth, A. S. & Tomaselli, G. F. Basic science of cardiac resynchronization therapy: molecular and electrophysiological mechanisms. *Circulation. Arrhythmia and electrophysiology* **5**, 594-603, doi:10.1161/CIRCEP.111.962746 (2012).
